# Depth inversion with a 3D structure influences brightness perception

**DOI:** 10.1371/journal.pone.0224192

**Published:** 2019-10-18

**Authors:** Tetsuya Arai, Tomohiro Masuda, Yuka Igarashi, Keiko Omori, Yasunori Aizawa, Naoe Masuda

**Affiliations:** 1 Faculty of Human Sciences, Bunkyo University, Koshigaya-shi, Saitama, Japan; 2 Faculty of Human Sciences, Kanagawa University, Yokohama-shi, Kanagawa, Japan; 3 College of Humanities and Sciences, Nihon University, Setagaya-ku, Tokyo, Japan; 4 Department of Functional Brain Imaging Research, National Institute of Radiological Sciences, National Institutes for Quantum and Radiological Science and Technology, Chiba-shi, Chiba, Japan; 5 Faculty of Letters, Keio University, Minato-ku, Tokyo, Japan; Universitat de Valencia, SPAIN

## Abstract

Whether or not depth perception influences brightness and/or lightness perception has been repeatedly discussed, and some studies have emphasized its importance. In addition, a small number of studies have empirically tested and shown the effect of depth inversion, such as seen in the Mach card illusion, on perceived lightness, and they interpreted such results in terms of lightness constancy. However, how *perceived brightness* changes contingent on depth inversion remains unexplained. Therefore, this study used the matching method to examine changes in brightness perception when depth inversion is observed. We created and used a three-dimensional (3D) concave object, composed of three sides made of card stock, which could be perceived as having two different shapes in 3D; it could be perceived as a horizontal concave object, corresponding to its actual physical structure, and as a convex standing object, similar in shape to a building. Participants observed this object as both a concave object and as a convex object, and judged the brightness of its surfaces during each observation. Our results show that the perception of the brightness of the object’s surfaces clearly changed depending on the perception of depth. When the object was seen as convex, one part of the surface was perceived as darker than when the object was seen as concave, but the other part of the surface remained unchanged. Here we discuss the relationship between depth perception and brightness perception in terms of perceptual organization.

## Introduction

While there are various definitions of brightness and lightness [1], brightness is often defined as a perceptional dimension corresponding to luminance that varies from dark to bright. Lightness is a perceptual dimension corresponding to the reflectance of an object that varies from black to white via gray.

One aspect of brightness and lightness studies is the relationship between brightness and/or lightness and depth perception. One of the most impressive experiments on that relationship was conducted by Gilchrist [2,3]. In Gilchrist’s experiments, the perceived surface changed from nearly white to nearly black only as a function of its perceived spatial position with no critical change in retinal image. These findings led to a coplanar ratio principle, according to which, luminance ratios between regions within the same depth plane are more significant in perceived lightness than ratios between regions within different depth planes. Similar ideas have also been proposed by Gestalt psychologists. For instance, Koffka [4] noted that coplanarity is an important grouping principle for lightness. Wolff [5] reported that a lightness contrast effect tended to occur when the plane was perceptually the same, and Kardos [6] found a small depth effect when a target was not adjacent to its coplanar neighbor. More recently, Radonjić, Todorović & Gilchrist [7] developed Gilchrist’s theory and the Kardos study [6], and observed a large depth effect on lightness without adjacency. In this way, effects of perceived depth on brightness or lightness (depth effects) were replicated with more sophisticated measurements, so that it is now believed that perceived arrangement can influence brightness and lightness perception.

Among these studies, some reported brightness and/or lightness changes with depth inversion. The original phenomenon was reported by Mach [8]. When the well-known Mach card illusion is observed, not only depth inversion but also brightness and lightness changes can be perceived. The original Mach card is a convexly bent card. When the left side of the card is illuminated slightly more brightly than the right side, we do not notice the difference in illumination. On the other hand, when we perceive the Mach card as concave rather than convex, the difference in brightness becomes clear. Though this phenomenon has been known for a long time, Mach [8] described it only qualitatively, and there are few empirical studies on this phenomenon that explore perceived 3D structure changes with the same retinal image. Beck [9] attempted to replicate the Mach card effect and demonstrated a weak effect of depth inversion on lightness (brightness in the original). Bloj, Kersten, and Hurlbert [10] showed a strong Mach effect using chromatic color, which was explained as being due to mutual illumination when the card was perceived in its concave shape. And, more systematically, Bloj and Hurlbert [11] tested the effects of depth inversion on lightness and brightness, and revealed that when an object was perceived as concave, there was no difference in lightness between the “light side” and “dark side”, but that the “dark side” was perceived as having low lightness when the object was perceived as convex. On the other hand, the difference in brightness between the “light side” and “dark side” was roughly the same for both convex and concave. They explained these results in terms of interpretation of illumination or lightness constancy. Although they concluded that the change in the perceived appearance of the surface with the depth inversion occurred only for lightness, their data (Experiment 1) indicated a slight difference in brightness perception between concave and convex conditions. That is, depth inversion may influence the perception of brightness as well as lightness when the same plane is compared. This is the first point of our study: In order to examine changes in brightness perception with depth inversion, not only the comparison between one surface and another surface but also the comparison between the same surfaces with or without inversion is required. Furthermore, as Adelson [12] and other studies demonstrated, changes in spatial configuration can result in differences in perceived surface brightness in two-dimensional (2D) displays, and in these studies, two types of figures with great difference in perceptual organization were compared. From these studies, it is possible to infer that the lack of influence of depth inversion on the perception of brightness in previous studies may be attributed to the changes of perceived structures being relatively small when observing inversion. Therefore, there is still room to examine whether depth inversion or change of 3D organization influence brightness perception with a novel observation object in which perceived structure can change substantially. This is the second point of our study. In order to embody the examination, some kind of “real object” is needed because pictorial models including computer graphics are insufficient to invoke the strong depth inversion discussed here.

The purpose of this study was to test the influence of depth inversion on perceived brightness using a 3D object that produces dramatic changes in how it is perceived structurally. Brightness changes were found in this setting, and we interpreted the results from perspectives other than that of lightness constancy discussed in previous studies.

## Experiment 1

Using a 3D object, we examined brightness changes when depth inversion was perceived. First, we tested whether or not participants could experience depth inversion with our original object. Then, we measured changes in perceived brightness by means of a matching method.

## Materials and methods

### Participants

Ten adults with normal vision served as participants (5 males and 5 females aged 20 to 32; mean age: 22.2 years). All participants could easily observe the depth inversion in the preliminary screening (shown below). We received prior approval for our study from the research ethics committee of Kanagawa University, and experiments were in accordance with the Declaration of Helsinki. We obtained informed consent in writing from participants before the experiment.

#### Stimulus and setting

**Fig 1:**
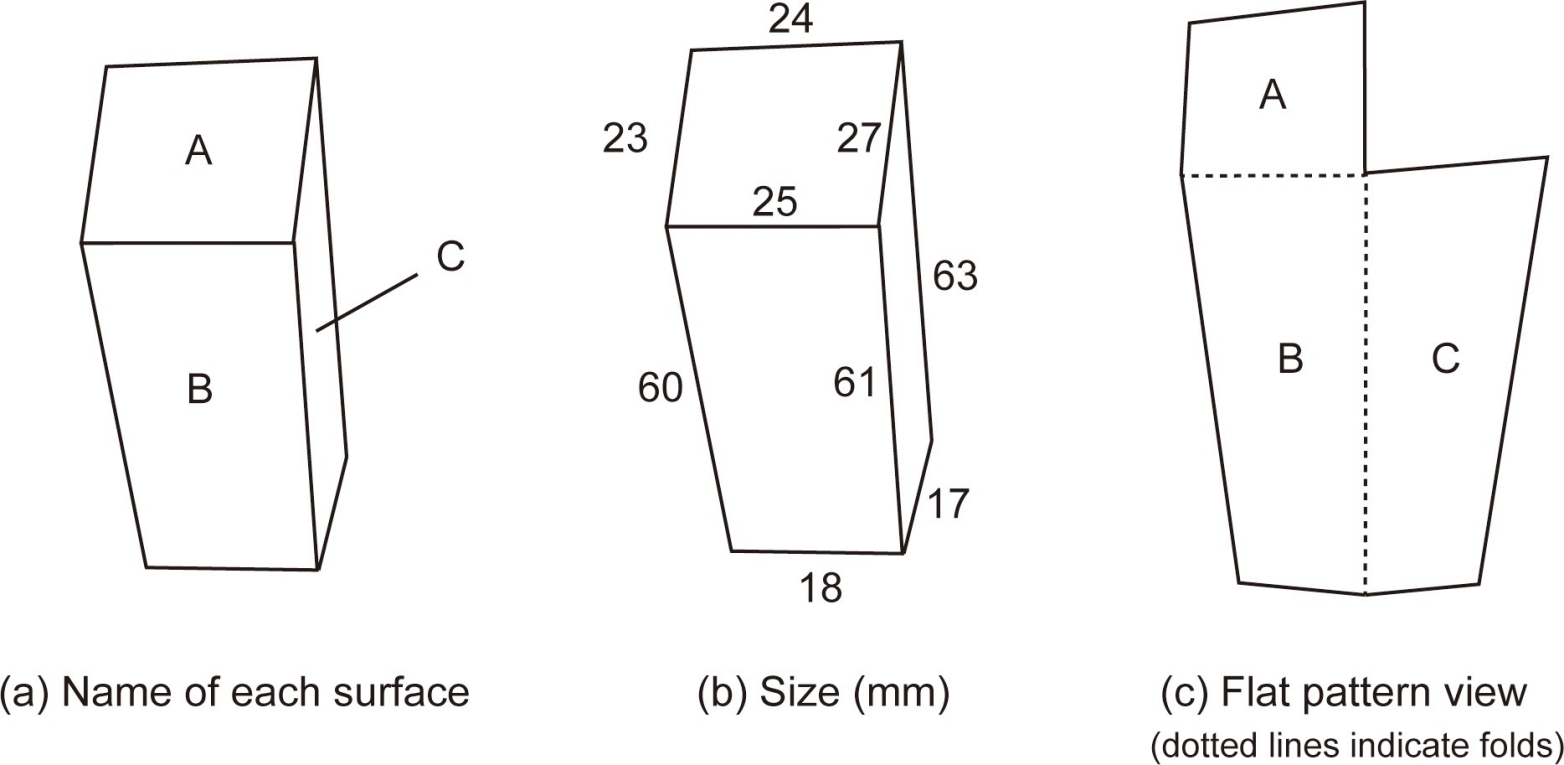
Schemas of the 3D object used in this study. Surface B is the bottom face and surfaces A and C are vertically oriented. Participants judged the brightness of surfaces A and B, but we did not use surface C as a target because it appeared very narrow in both conditions.

**Fig 2:**
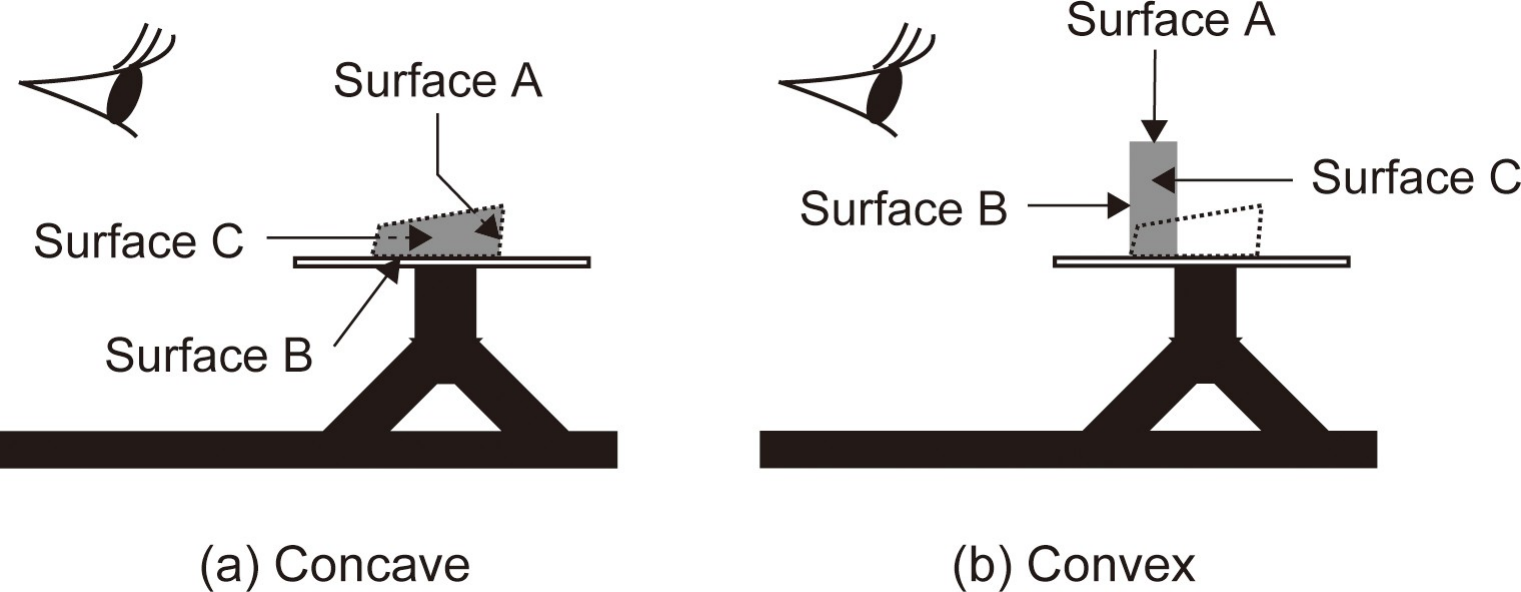
Schemas of how participants perceived the stimulus pattern. The dotted quadrangle shows the actual position and the gray one shows the perceived position. In the concave condition, all surfaces are observed as inside faces of the object and it is seen as horizontal and concave, corresponding to its actual structure. In the convex condition, all surfaces are observed as outside faces of the object and it is seen as vertical and convex, similar to the shape of a building (see also Fig 1).

We created a 3D concave object composed of three faces by cutting and folding gray card stock (Fig 1). When we casually look at the object, it appears as a horizontal concave object, which corresponds with its actual physical structure. When we continue to observe it, we may experience an inversion of depth after which the object appears as a standing convex object, similar in shape to a building. This observation tends to occur when one eye is covered. The important point of this object is that the perceived change in the structure is greater than that for the Mach card. In the Mach card, the “apparent” angle between the illumination and each face remains almost unchanged even when inverted, but in our setting, that angle dramatically changes from vertical to horizontal or from horizontal to vertical (Fig 2).

The Munsell value and luminance value of each surface is shown in Table 1 (the background was N 9.2, 83 cd/m^2^). We had three different objects corresponding to three experimental conditions for lightness value; in each condition, the lightness value of surface A was equal to that of surface C, and that of surface B was higher than both A and C in order to examine the influence of absolute lightness values. Additionally, the lightness value of surface B was higher than that of A and C in order to clarify the difference in appearance between surfaces A and B. Surface A was placed in a frontal parallel plane and surface B was the bottom face. A chin rest was used to fix the participants' viewing distance at 40 cm from surface A, and the object was observed at an angle of 30 degrees of depression. Each surface and matching scale was illuminated by dimmable fluorescents (Illuminant D65) with an illuminance of 400 lx. The three lights were arranged in a U shape with one above the participant’s head, another to the upper left, and the third above and in front of the participant, so as to illuminate the object almost uniformly. The light source was invisible to the participants while they were observing the object in order to prevent them from inferring the direction of the illumination, and the positioning of the lighting was not included in our explanation to them. Since participants may use shadows cast by the object as cues to judge lightness, we adjusted the position of the light source and the object so that the shadow was cast behind the object and out of view. Bloj and Hurlbert [11] reported that when the cast shadow was removed, lightness constancy for the Mach card was not satisfied and one side of the card appeared lighter than the other. This means that, for our experiment, removal of the cast shadow should counteract lightness constancy and any difference in Munsell matches should indicate a difference in brightness.

**Table 1:** Actual Munsell values and luminance values (cd/m^2^) of surface.

	Surface		
Lightness-value condition	A	B	C
1 (low)	4.2 (14)	5.3 (22)	4.2 (14)
2 (medium)	5.7 (27)	7.0 (43)	5.7 (27)
3 (high)	7.2 (45)	8.7 (72)	7.2 (45)

Surfaces A and C had the same values, and surface B was lighter than surfaces A and C in all lightness-value conditions.

### Procedure

#### 1) Observation of depth inversion

Participants observed the object with their chins on the chin rest and they were not allowed to move their head during observation. First, they were instructed to observe the object and to report freely how it appeared. All participants reported that the object was concave and some of them reported that it also appeared convex. Second, they did the same observation with their left eye covered with an eye occluder and all of them reported that the object appeared convex and standing like a building (Fig 2, right). To confirm that participants saw the shapes which we expected, they were asked to compare the convex appearance of the object with a real, convex, rectangular, solid object and we thus confirmed that they understood the difference between the two depth conditions. Since all participants experienced the depth inversion, they all took part in the next brightness-matching task.

#### 2) Brightness-matching task

At the beginning of each session, participants were instructed to observe a stimulus pattern with the object either convex or concave, and they continued as instructed through one session of 15 trials: five repetitions of three lightness-value conditions. Use of the eye occluder was optional for the convex condition, but all participants used it. They were instructed to observe the entirety of a stimulus pattern and to choose the patch from the matching scale that most closely matched the brightness of surfaces A and B. We did not use surface C as a target because it appeared very narrow in both conditions. Participants used the same eye(s) both when observing the object and when observing matching patches, and the brightness was compared for the same eye(s) (e.g., right and right; both and both) and not for different eyes (e.g., both and right). The matching scale consisted of 12 circle patches ranging from N 3.5 to N 9.0 of the Munsell color system (0.5 steps) and was placed to the left of the object out of the field of view of participants while observing the object. We instructed participants not to judge the nature of the surface or the shade of gray of a surface (i.e., lightness), but rather to judge the appearance of the overall light intensity reflected off a surface (i.e., brightness) by explaining, in Japanese, as follows: “Please judge the apparent nature of each surface. Though the dark part of the object may be seen as a shadow or stain, do not infer the physical nature of the surface by subtracting shadow or stain.” After the brightness matching tasks, we asked participants how they had made their judgments in order to confirm that they had judged brightness, not lightness. Each participant underwent four sessions: two for the convex condition and two for the concave condition. The order in which participants made brightness matches in one session and among the four sessions was counterbalanced.

### Results and discussion

**Fig 3:**
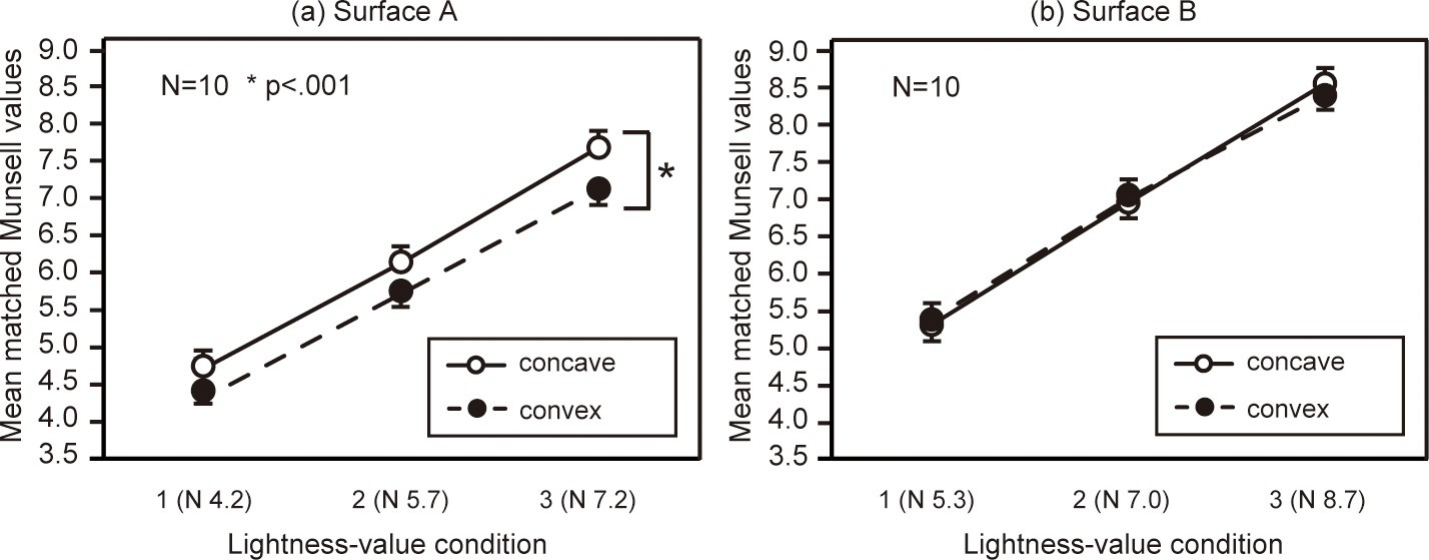
**Participants’ matches to surfaces A and B in Experiment 1.** Each data point represents the mean matched Munsell value for each respective condition, and error bars show standard error.

Fig 3 summarizes participants’ brightness matches for surfaces A and B. Surface A was perceived as darker in the convex condition than in the concave condition. For surface B, there was no difference between convex and concave conditions. A three-way repeated-measures ANOVA showed that all the main effects were significant (surface: *F*(1,9) = 204.82, *p <* .01, inversion: *F*(1,9) = 22.24, *p <* .001, lightness value: *F*(2,18) = 1598.55, *p <* .001). The interaction between surface and inversion was significant (*F*(1,9) = 20.33, *p <* .01), and there was a simple main effect of inversion on surface A (*F*(1,18) = 42.12, *p <* .001).

**Fig 4:**
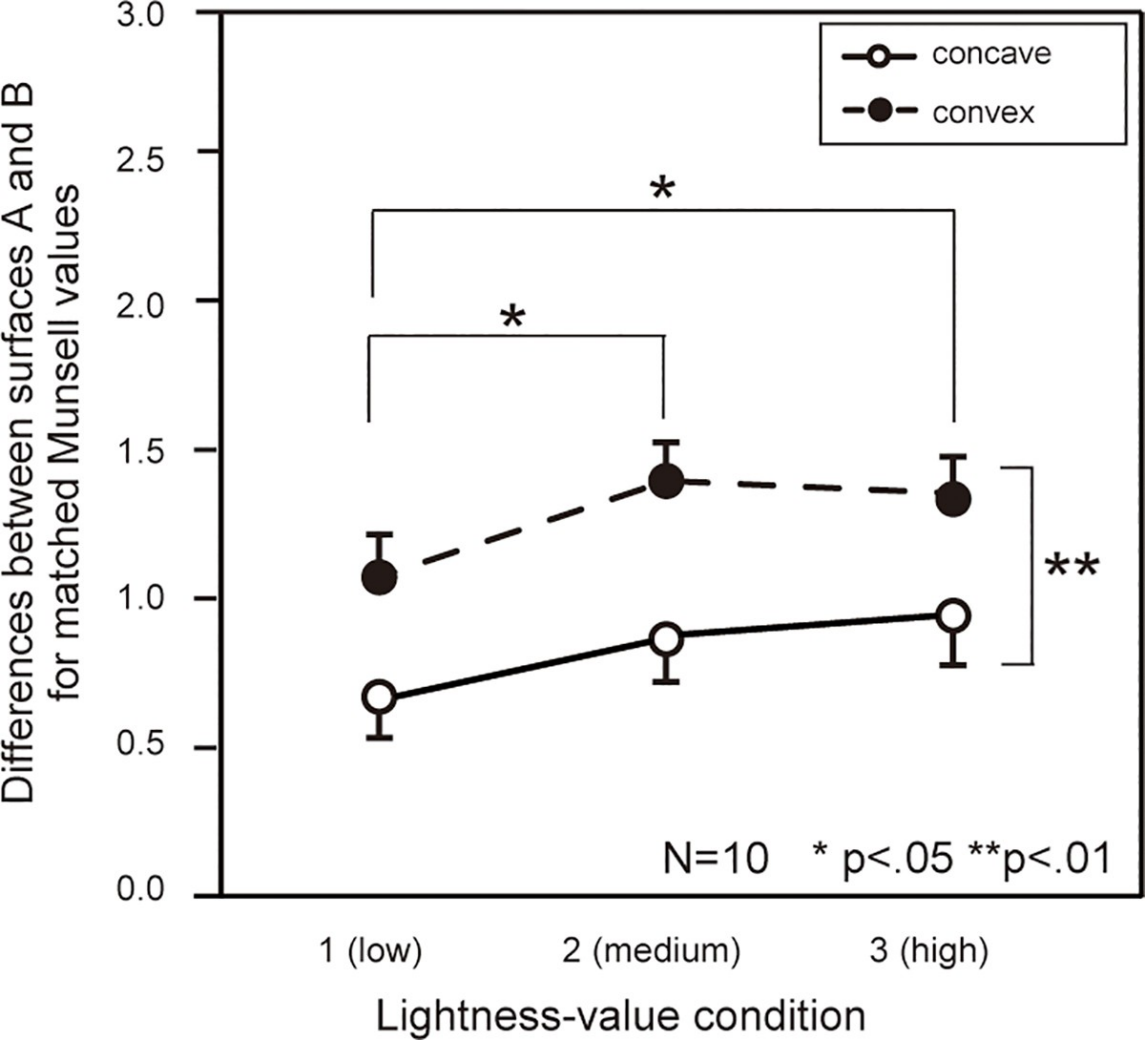
**Differences between surfaces A and B for matched Munsell values in Experiment 1.** We took the differences between the data for surfaces A and B shown in Fig 3. Error bars show standard error.

It was not surprising that surface B was perceived as brighter than surface A because the lightness values of surface B were higher than those of surface A; however, the difference in brightness between both surfaces appeared to be greater in the convex condition. To make clear the relationship between surfaces A and B, the differences in matched brightness for each surface were analyzed (Fig 4). Differences in the brightness between surfaces A and B were significant for the convex condition. However, the effect was relatively small for the low lightness-value condition. A two-way repeated-measures ANOVA showed that all main effects were significant (lightness value: *F*(1,9) = 8.47, *p <* .01, inversion: *F*(1,9) = 20.33, *p <* .01), and multiple comparisons revealed there were significant differences between lightness-value 1 and 2 as well as between conditions 2 and 3 (*p* < .05). This means that the depth effect was smaller in the low lightness-value condition.

With Experiment 1, we showed that depth perception can influence perceived brightness. However, the extent of this influence was different among lightness-value conditions. Kozaki [13] emphasized that not only the reflectance of the inducing field, but also that of the background influenced perceived brightness in contrast and assimilation. So, it is possible that the smaller influence of depth perception in lightness-value condition 1 was caused by the greater contrast between the object and the background. In the second experiment, we tested that hypothesis by varying the lightness values of the background.

## Experiment 2

To test the influence of the background on brightness perception, we varied the lightness values of the background used in Experiment 1.

## Materials and methods

### Participants

Ten adults, three of whom also participated in Experiment 1, with normal vision served as participants (5 males and 5 females aged 20 to 28; mean age: 21.9 years). All could easily observe the depth inversion in the preliminary observation. We received prior approval for our study as with Experiment 1 and obtained informed consent in writing from participants before the experiment.

#### Stimulus and setting

The same settings were used as for Experiment 1 except for the object and background reflectance. Rather than three lightness-value conditions, we used only the first lightness-value condition (surface A: N4.2, surface B: N5.3) from Experiment 1 and we added three background conditions. Background 1 (Bg1) was black and much darker than the object (N 1.9), background 2 (Bg2) was slightly lighter than the object (N 5.4), and background 3 (Bg3) had the same Munsell value as that of Experiment 1 (N 9.2), making it lighter than the object.

### Procedure

Instructions and other procedures were the same as for Experiment 1.

### Results and discussion

**Fig 5:**
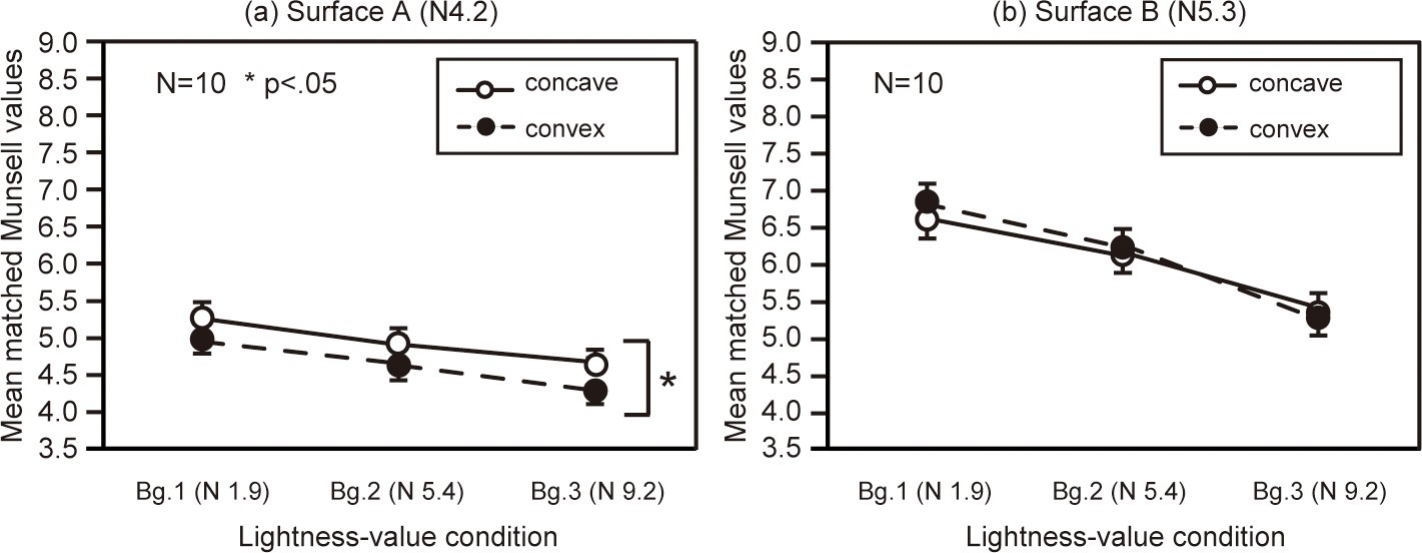
**Participants’ matches for surfaces A and B in Experiment 2.** Each data point represents the mean matched Munsell value for each respective condition, and error bars show standard error.

**Fig 6:**
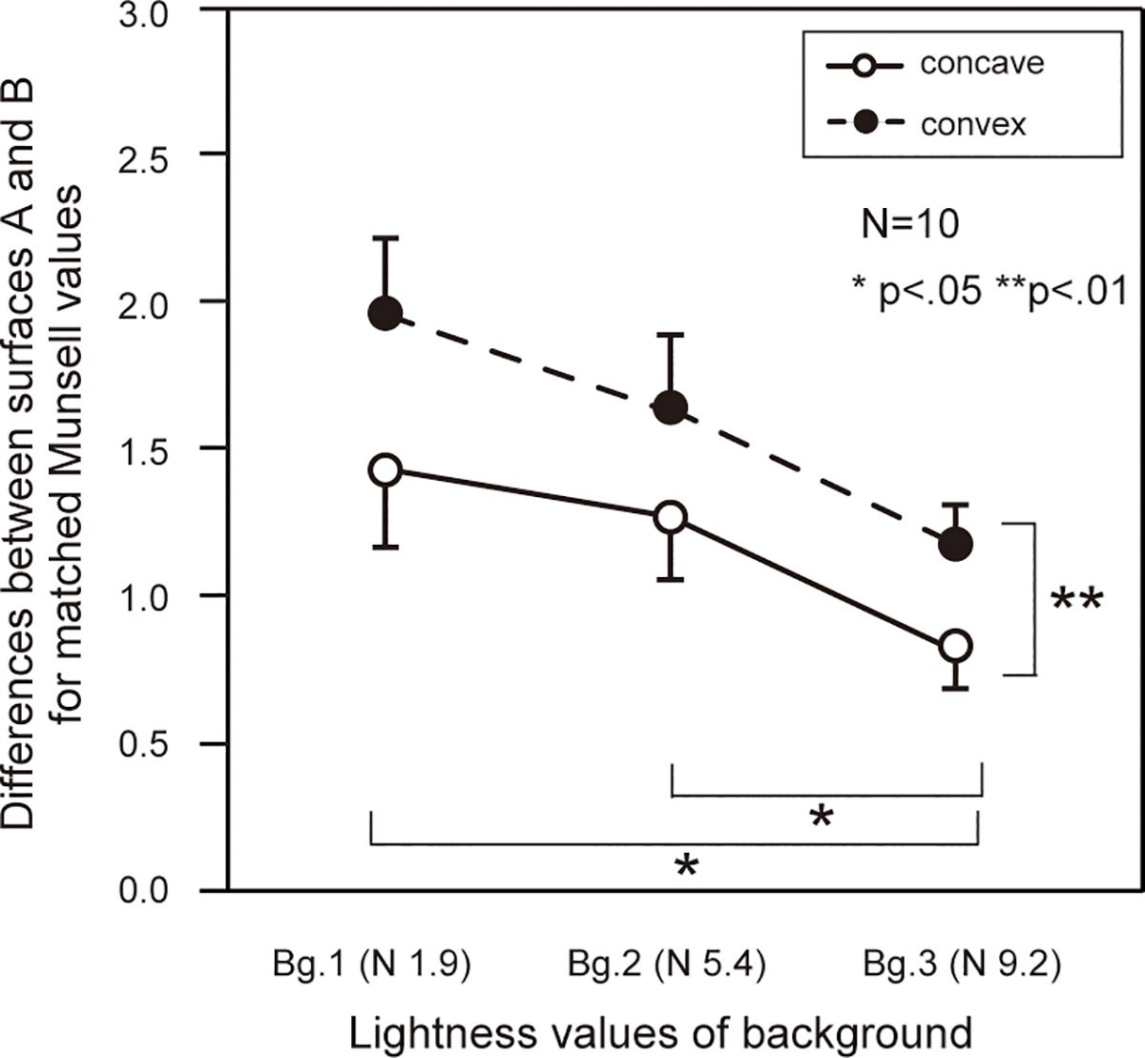
**Differences between surfaces A and B for matched Munsell values in Experiment 2.** We took the differences between the data for surfaces A and B shown in Fig 5. Error bars show standard error.

Tendencies of participants’ judgments were similar to those of Experiment 1; that is, surface A was perceived as darker in the convex condition than in the concave condition, and surface B in the convex condition was perceived as almost equal to that in the concave condition (Fig 5). A three-way repeated-measures ANOVA showed that the main effects of surface (*F*(1,9) = 74.28, *p <* .001) and lightness value of the background (*F*(2,18) = 54.99, *p <* .001) were significant. The interaction between surface and inversion was also significant (*F*(1,9) = 12.14, *p <* .01), and there was a simple main effect of inversion on surface A (*F*(1,18) = 7.37, *p <* .05). As for the difference between surfaces A and B (Fig 6), a two-way repeated-measures ANOVA showed a main effect of inversion (*F*(1,9) = 12.14, *p <* .01) and lightness value (*F*(2,18) = 14.14, *p <* .001), and there were significant differences between Bg1 and Bg3 and between Bg2 and Bg3 (*p <* .05). Therefore, as the lightness value of the background increased, the effect of inversion decreased, explaining why the depth effect was relatively small in lightness-value condition 1 of Experiment 1.

## General discussion

Whereas the depth effect on lightness has been shown, that on brightness has not [11]. In this study, however, changes in the perceived shape of a 3D structure influenced brightness perception. As mentioned above, a larger change in perceived depth could be experienced with our stimuli than observed in previous studies, and it is possible that such perceived structural change affected perceived brightness as discussed below.

The depth effect on lightness has been discussed within the framework of lightness constancy [7,11,14], in which depth inversion can change perceived illumination and collapse constancy. For example, when the Mach card is perceived as convex, the difference between the dark side and the light side is interpreted as the difference in illumination and the lightness of both sides is judged the same (lightness constancy). On the other hand, when it is perceived as concave, different reflectance is assigned to each side and lightness constancy fails. Regarding this point, we were careful to avoid shadows in the stimulus set-up, and since shadows are the primary factor for lightness constancy according to Bloj and Hurlbert, we are confident that any difference in Munsell matches must be due to brightness difference rather than lightness. This shows that we need to interpret the depth effect on brightness from perspectives other than constancy. One possible interpretation is from the viewpoint of perceptual organization. The change of perceived depth caused a change in the perceptual organization of the apparent surface shape and apparent illumination. In the concave condition, it is possible that surfaces A and B were perceived as homogeneously illuminated surfaces and grouped depending on the common illumination [15,16], and the brightness of each was relatively approximate. In the convex condition, each surface may have been perceived as differently illuminated, resulting in a greater difference in their perceived brightness. A similar interpretation is found for the Koffka ring [4]. With the Koffka ring, when a contiguous gray ring is placed on a bicolored background (half white, half black), the ring appears to be a homogeneous gray. However, when the ring is divided by a bar along the border between the two colors of the background, the two halves of the ring appear to be different shades of gray. The half of the ring on the black background appears lighter than the half of the ring on the white background. Koffka [4] attempted to explain the phenomenon in terms of “forces of cohesion” which hold the ring together. In a similar way, the perceptual division of surfaces A and B in our experiment may have led to different perceptions of brightness. In the concave condition, both surfaces A and B (and surface C) were coherent in common illumination, and the perceptual organization yielded approximation of brightness like the assimilation effect found by Fuchs [17]. On the other hand, in the convex condition, such organization of the shape of the surfaces collapsed and the brightness of surfaces A and B were differentiated in correspondence to their luminance. This interpretation may be corroborated by considering the following: In Experiment 1, the ratios between the actual Munsell values of surfaces A and B (A divided by B) were 0.79, 0.81 and 0.83, respectively, for each lightness-value condition, and the perceived values were 0.87, 0.88, and 0.89, respectively, for the concave condition and 0.81, 0.81, 0.84, respectively, for the convex condition. Since the relationship between surfaces A and B in the convex condition is more similar to the actual physical relation than that in the concave condition, it seems that the perceived brightness change that occurred with the change in perceived depth mainly occurred in the concave condition rather than in the convex condition.

## Conclusions

In this study, we examined the influence of depth perception on brightness perception and demonstrated that brightness changed depending on the 3D structure perceived. Our study used a 3D object and demonstrated an effect of depth on brightness that has only ever been examined using 2D displays. This approach has the potential to reveal characteristics of brightness perception in the natural world.
